# Recent advances in the understanding and management of primary hyperparathyroidism

**DOI:** 10.12688/f1000research.21569.1

**Published:** 2020-02-25

**Authors:** Melanie Goldfarb, Frederick R. Singer

**Affiliations:** 1 Center for Endocrine Tumors and Disorders, John Wayne Cancer Institute at Providence Saint John’s Health Center, Santa Monica, CA, 90404, USA; 2 Endocrine/Bone Disease Program, John Wayne Cancer Institute at Providence Saint John’s Health Center, Santa Monica, CA, 90404, USA

**Keywords:** primary hyperparathyroidism

## Abstract

Primary hyperparathyroidism is a hormonal disorder whose prevalence is approximately 1–2% in the United States of America. The disease has become more recognizable to clinicians in an earlier phase and, at present, patients can be diagnosed with “classic”, “normocalcemic”, “normohormonal”, or “mild, asymptomatic” primary hyperparathyroidism. Surgery, with a focused parathyroidectomy when possible, or a four-gland exploration, is the only way to cure the disease. Cure is determined by use of intra-operative parathyroid hormone monitoring with long-term cure rates ranging from 90–95%. Newer adjuncts to surgery include CT or PET imaging and near-infrared immunofluorescence. This article highlights updates in parathyroid disease and advances in parathyroid surgery; it does not provide a comprehensive summary of the disease process or a review of surgical indications, which can be found in the AAES guidelines or NIH Symposium on primary hyperparathyroidism.

## Introduction

Primary hyperparathyroidism (PHPT) is a hormonal disorder whose prevalence is approximately 1–2% in the United States of America and is less prevalent in countries where biochemical screening is less common. It is most often diagnosed in postmenopausal women, though prevalence has been reported to be as high as 0.5–1% in older men. Recognition of this problem more than 90 years ago was originally in patients with severe symptomatology, such as impaired cognition, peptic ulcers, constipation, muscle weakness, and arthralgias, with the first parathyroidectomy carried out by Sir John Bland-Sutton before 1917 ^1^. Previously, Felix Mandl of Germany had long been credited with performing the first parathyroidectomy in 1926 ^2^. Over the years, as biochemical, pathological, and radiological technology has evolved, the clinical state of patients has changed radically. Initially, serum calcium and parathyroid hormone (PTH) levels were markedly elevated with kidney stones and fractures prominent. Over time, PHPT became more recognizable to clinicians in an earlier phase as routine chemistry became widely used and osteoporosis diagnosis increased with the advent of bone densitometry ^3^. At present, a significant number of patients have “no symptoms”, although bone density may be decreased and occult nephrolithiasis may be present ^4– 6^. Older adults are frequently underdiagnosed and undertreated, perhaps because of a lack of understanding of the consequences of the disorder ^7^. A large survey of primary care patients has concluded that health cost savings associated with fracture risk could be achieved by appropriate screening and treatment of patients with a life expectancy of 16 years ^8^.

## Classification of primary hyperparathyroidism

PHPT is not a single entity. In most normal subjects, four glands are present and one or more may become pathologic. Other endocrine disorders may be present depending upon genetic abnormalities, which may be inherited. Table 1 summarizes the types of PHPT that have been reported. A single parathyroid adenoma is found in about 80% of cases. Double adenomas and sporadic hyperplasia account for the majority of the remaining cases, and all the other multi-gland cases account for the rest. Parathyroid carcinomas are rare.

**Table 1.** Classification of primary hyperparathyroidism.

**Parathyroid adenomas** Normohormonal and normocalcemic subsets Double parathyroid adenomas Hyperparathyroidism-jaw tumor syndrome Familial isolated hyperparathyroidism Parathyroid carcinomas **Parathyroid hyperplasia** Sporadic Normocalcemic and normohormonal subsets Multiple endocrine neoplasia Type 1: primary hyperparathyroidism, pancreatic neuroendocrine tumors, pituitary adenomas Type 2A: primary hyperparathyroidism, medullary thyroid carcinoma, pheochromocytoma Type 2B or 3: medullary thyroid carcinoma, pheochromocytoma, ganglioneuromas, marfanoid habitus, primary hyperparathyroidism (uncommon) Type 4: primary hyperparathyroidism, pituitary tumors Familial isolated hyperparathyroidism Neonatal severe primary hyperparathyroidism Parathyromatosis Lithium

Unlike classical PHPT, some patients are normocalcemic despite elevated serum PTH and others are hypercalcemic with serum PTH levels in the normal range ^9^. In a 573-patient study of patients with PHPT, 405 patients were classic (70.7%), 96 were normohormonal (16.8%), and 72 were normocalcemic (12.5%) ^10^. Normocalcemic hyperparathyroidism was associated with multi-gland disease (MGD) in 45% of patients, but only 10% in the normohormonal group and 9% in classic PHPT. Twelve months after surgery, the biochemistry was normal in >98% in all three groups.

It is important to distinguish PHPT from the rare disorder familial hypocalciuric hypercalcemia, of which there are three subtypes: FHH1, FHH2, and FHH3 ^11^. Generally, these require no treatment. The diagnosis can be achieved by measuring 24-hour urinary calcium excretion. A calcium/creatinine level of less than 0.01 is found in most patients. Serum PTH levels are usually normal but may be elevated, and serum magnesium is usually elevated. These autosomal dominant disorders are caused by a loss-of-function mutation in the calcium-sensing receptor gene ( *CaSR*) in the case of FHH1, loss of function in G-protein subunit alpha-1 (GNA11) in FHH2, and loss of function in adaptor protein-2 sigma 1 subunit (GNA11) in FHH3.

## Indications for parathyroidectomy

Today, parathyroidectomy is recommended for the majority of patients diagnosed with PHPT. National and international guidelines recommend the minimum indications for surgical treatment of PHPT, but ultimately it is a decision reached by the patient, surgeon, and endocrinologist ^12, 13^. Surgery remains the most cost-effective long-term strategy, even in mild, asymptomatic patients, when incorporating fracture risk, in addition to decreasing cardiovascular risk factors and kidney stone formation and producing sustained benefits in quality of life (QOL) and neurocognition ^14– 19^. Frailty, based on either co-morbid conditions or an actual frailty score, not age itself, appears to be the main contra-indication for surgery, since parathyroidectomy can be beneficial even in the very elderly ^20, 21^. For patients who refuse, cannot medically have surgery, or who are not cured by surgery, cinacalcet, an inhibitor of the calcium-sensing receptor, can produce long-term normocalcemia ^22^. A bisphosphonate can also be added to prevent bone loss.

Recent QOL studies using either older scoring measures or a new PHPT-specific health-related QOL questionnaire, PHPQoL, have demonstrated decreased symptomatology and recovery not only for patients with classical PHPT but also in patients with normocalcemic and mild PHPT ^23– 27^. Improvements in QOL factors were measured as early as the first week after surgery and were sustained long term in areas of mental health, sleep quality, somatic and cognitive depression domains, and neurocognition. The updated AAES management guidelines cite neurocognitive symptoms as another surgical indication in addition to kidney stones, osteoporosis, decreased kidney function, and hypercalcemia that are dictated in the NIH guidelines.

## Pre-operative imaging

Imaging for parathyroid localization should be performed only after the diagnosis of PHPT has been confirmed *and* the decision has been made to proceed with surgery. Localization is *not* to be used for diagnosis and should *not* be used to decide if a patient should proceed with surgery. Moreover, the old adage still holds true: the best localization is an experienced parathyroid surgeon. With that said, the primary role of pre-operative imaging is to decide if a patient is a candidate for a focused, minimally invasive parathyroidectomy.

Ultrasound (US) is the most cost-effective and a fairly sensitive localization technique and should be the first imaging test ordered for a patient. Surgeon-performed US has the greatest sensitivity and should be standard for any surgeon performing parathyroidectomies, in both the clinic and the operating room. US is best at detecting enlarged inferior glands, and understandably less so for superior glands owing to their location behind the thyroid gland or ectopic glands. If the US is negative or equivocal, in the United States, a CT scan is the most sensitive and cost-effective first- or second-line localization test ^28, 29^. ****Sestamibi remains inferior because of the false positive rates with nodules and especially thyroiditis, which are present in many individuals ^30^. Although some centers report almost equivalent efficacy with a two-phase compared to four-phase CT scans, the most common technique is a three-phase scan ^31^. No imaging, including CT scans, has a high accuracy in patients with MGD ^32– 34^. In the re-operative setting, multimodal imaging with at least two of Sestamibi, CT, MRI, and US has improved successful localization to 91.6% in one study of almost 350 patients ^35^.

### Newer imaging techniques

Though not traditionally used for localization of parathyroid adenomas, dynamic MRIs, with or without ^18^F PET, have been trialed by some institutions and demonstrated reasonable sensitivity and positive predictive value (PPV) ^36– 38^. Mayo clinic has trialed a novel radiotracer, carbon-11-choline, with PET/CT. In a small study, it had 100% sensitivity for the detection of abnormal single parathyroid glands, including abnormal glands as small as 6 mm or 50 mg. Unlike other nuclear medicine scans, imaging was completed in only 15 minutes and the radiation dose was lower than that of 4D CT ^39^.

## Parathyroid surgery

Parathyroidectomy, regardless of the surgical technique (minimal access, video-assisted, robotic, or transoral) and whether it is a focused or a bilateral neck exploration (BNE), can be performed as an outpatient procedure. If the abnormal parathyroid gland(s) can be localized pre-operatively, a focused approach can be performed, resulting in decreased operative and anesthesia time, incision size (usually), and the amount of dissection, and therefore scar tissue, in the patient. In both the primary and the re-operative setting, parathyroidectomies should be performed by a high-volume surgeon and a multidisciplinary team engaged for complicated cases ^12, 40, 41^. When parathyroidectomy is performed by high-volume surgeons, rates of nerve injury are low, but post-operative hypercalcemia and hypocalcemia rates are reported to be 5% for index parathyroidectomies and 10% for re-operative parathyroidectomies ^42^.

### Determination of “cure”: intraoperative parathyroid hormone

Intra-operative PTH (IOPTH) continues to be the gold standard for confirmation of “cure” in the operating room. PTH has a half-life of 3.5 minutes that allows almost immediate evidence of a cure after removal of the abnormal inciting gland(s). IOPTH continues to add value in upwards of 15–20% of parathyroidectomies, depending on the patient cohort ^27, 43^. However, even with localized imaging and a significant IOPTH fall well into the normal range, there is a 2–3% failure rate for unexplained reasons. Most commonly, IOPTH levels are drawn pre-incision, at 0, 5, and 10 minutes; other variations include levels at 0, 5, and 15 minutes or at 5, 10, and 20 minutes. The Miami criteria state that a 50% decrease from pre-operative or pre-excision PTH levels at 10 minutes correlates with a cure.

Further refinement of the criteria dictates that the final IOPTH level falls into the “normal” range, and some studies report improved cure rates when final IOPTH levels fall below 40 ^44^. Baseline PTH does not seem to affect cure rate if the >50% drop is achieved, which is important given the increased number of patients with “normohormonal” PHPT having surgery ^45^. Patients who have been on long-term bisphosphonates or certain other medications or have a decreased creatinine clearance may have different PTH kinetics, taking 20–30 minutes or longer to fall to its final value and/or require stricter criteria to declare a “cure”, though there is debate with regard to the extent of chronic kidney disease that causes altered PTH kinetics ^46– 48^.

### Prediction of multi-gland disease

As the definition and surgical referral of PHPT has expanded over the past decade or so, a greater percentage of patients, possibly as high as 25%, appear to have MGD, either double adenomas or four-gland hyperplasia. As mentioned above, pre-operative imaging is notoriously poor at recognizing and localizing MGD ^32, 43^. Currently, IOPTH is the best test surgeons have for determining the presence of MGD. However, experiments with machine learning report a 94.1% accuracy, 94.1% sensitivity, 83.8% specificity, and 94.1% PPV for predicting MGD ^49, 50^. Patients with normocalcemic PHPT, normal baseline PTH, and mild biochemical profiles appear to have higher rates of MGD ^10, 51– 53^.

### Long-term outcomes

Historically, after surgical parathyroidectomy for “classic” PHPT, long-term cure rates were reported at close to 95% after either a focused parathyroidectomy or BNE. However, more recent long-term follow-up studies of more mixed PHPT populations with mild, normocalcemic, and normohormonal disease report that recurrence rates may be as high as 10% at 15+ years ^54^. The number of glands removed at the initial operation does not appear to impact recurrence rates, although double adenomas may have higher rates of persistent disease immediately after surgery ^55^. A recent study reported that besides the final IOPTH, patients with 6-month calcium levels <9.7 and concomitant normal PTH had the best long-term cure ^54^. Following surgery, PTH may remain elevated in up to 25% of patients who are cured from parathyroidectomy, which does not impact long-term cure rates and is more common in patients with pre-operative vitamin D deficiency and impaired renal function ^56^. Additionally, up to 10% of patients experience a delay in normalization of their calcium levels for up to two weeks, which also does not appear to impact rates of recurrent or persistent disease ^57^.

### Parathyroid autofluorescence: a new intra-operative adjunct?

Intrinsic near-infrared (NIR) autofluorescence may improve intraoperative identification of both normal and abnormal parathyroid glands. This could negate the need for a frozen section during surgery to confirm parathyroid tissue, if the technology is proven, as well as potentially nullify the need for prophylactic thymectomy during four-gland explorations when an inferior gland has not been identified and the IOPTH remains elevated. In the past few years, single institution studies have reported on their positive experiences with mostly fiber-based probe technology. Sensitivity, specificity, PPV, and negative predictive value (NPV) were as high as 95.8%, 97.2%, 95.1%, and 99.1%, respectively, in one study for detecting or confirming parathyroid tissue, and the addition of NIR helped to identify the abnormal gland in 20% of PHPT patients as well as rule out soft tissue as not parathyroid in another 15% of patients with PHPT ^58, 59^. Trials that combined NIR with injections of either methylene blue or indocyanine green did not seem to provide additional benefit ^60– 63^. Only one study thus far has looked at the different autofluorescence in hyperfunctioning versus normal parathyroid glands and reported that hyperfunctioning glands had a lower mean autofluorescence with a more heterogeneous pattern than normal parathyroid glands ^64^.

### Updates in special patient populations

***MEN1.*** A few centers have recently reported their experience with performing a less-than-subtotal (SPTX) or total (TPTX) parathyroidectomy for MEN1 patients. Given the high rate of post-operative hypocalcemia from those two procedures, especially TPTX, which can be debilitating, as well as the high rate of recurrent disease requiring reoperation after SPTX, a more limited initial approach seems reasonable in select patients ^65– 67^. Patients with positive pre-operative localization would be offered a more focused approach, whether single-gland or unilateral (i.e. two gland) excision, and would be counseled on the likely need for another operation at a future date. It would not be a classic “re-operation” at that time given that all parathyroid gland(s) would be removed in any explored quadrant at the time of the primary operation ^68^. Of note, in a small study, autofluorescence does not appear to be a useful adjunct during surgery in these patients ^69^.

***Pregnant females.*** Two published studies report that not only is parathyroidectomy during pregnancy safe and effective, but it also provides improved fetal and maternal outcomes compared to medical management during gestation ^70, 71^.

***Parathyromatosis.*** Parathyromatosis is a rare cause of hyperparathyroidism ^72^. Type 1 is thought to be of embryonic origin and consists of multiple nodules of benign hyperfunctioning parathyroid tissue, mainly consisting of chief cells, scattered throughout the neck and superior mediastinum. Type 2 occurs mainly after parathyroid surgery in patients with parathyroid hyperplasia accompanying chronic renal failure. It is a consequence of gland fracture and autotransplantation of parathyroid tissue in the neck at the time of parathyroid surgery. Surgical cure is difficult, and patients may require cinacalcet therapy and a bisphosphonate for management.

***Lithium-treated patients.*** The prevalence of hypercalcemia in lithium-treated patients is very high, and the long-term surgical cure rate is <50%, with most patients displaying hyperplasia on pathology ^73^.

## Summary

PHPT is prevalent, although older adults are frequently underdiagnosed and undertreated, perhaps owing to a lack of understanding of the consequences of the disorder. We now recognize multiple phenotypes of the disease that do not all include significantly high calcium and PTH levels. Advances in pre-operative imaging and surgical adjuncts continue to advance our ability to cure the disease, but more research into the genetic and molecular landscape of the disease is needed to improve our understanding and disease cure rates.
